# Combined analysis of IGHV mutations, telomere length and CD49d identifies long-term progression-free survivors in TP53 wild-type CLL treated with FCR-based therapies

**DOI:** 10.1038/s41375-021-01322-1

**Published:** 2021-06-19

**Authors:** Andrea G. S. Pepper, Antonella Zucchetto, Kevin Norris, Erika Tissino, Jerry Polesel, Zarni Soe, David Allsup, Anna Hockaday, Pei Loo Ow, Peter Hillmen, Andrew Rawstron, Daniel Catovsky, Pietro Bulian, Riccardo Bomben, Duncan M. Baird, Christopher D. Fegan, Valter Gattei, Chris Pepper

**Affiliations:** 1 Brighton and Sussex Medical School, University of Sussex, Brighton, United Kingdom; 2 Clinical and Experimental Onco-Hematology Unit, Centro di Riferimento Oncologico di Aviano (CRO) IRCCS, Aviano, Italy; 3 Division of Cancer and Genetics, School of Medicine, Cardiff University, Heath Park, Cardiff, United Kingdom; 4 Unit of Cancer Epidemiology, Centro di Riferimento Oncologico di Aviano (CRO) IRCCS, Aviano, Italy; 5 Leeds Teaching Hospital Trust, Leeds, United Kingdom; 6 Hull York Medical School, University of Hull, Hull, United Kingdom; 7 Clinical Trials Research Unit, Leeds Institute of Clinical Trials Research, University of Leeds, Leeds, United Kingdom; 8 Section of Experimental Haematology, Leeds Institute of Medical Research at St James’s, University of Leeds, Leeds, United Kingdom; 9 Institute of Cancer Research, Sutton, United Kingdom

**Keywords:** Translational research, Chronic lymphocytic leukaemia

## To the Editor:

Although there has been a revolution in the treatment of chronic lymphocytic leukemia (CLL), in the absence of curative therapy, the challenge of precision medicine remains the same: to give the right drugs to the right patients, at the right time [1, 2]. In this context, one of the key questions is whether there is still a role for chemoimmunotherapy (CIT). In this study, following the REMARK criteria (Supplementary Table 1), we took advantage of samples and outcome data derived from CLL patients enroled in two different UK clinical trials (ARCTIC/ADMIRE, Supplementary Table 2), treated in the frontline setting with comparable FCR/FCR-like regimens (Supplementary Fig. 1) [3, 4]. We demonstrated that only a minority of CLL patients achieved long-term progression-free survival (PFS), reliably identifiable by combining TP53 disruption status, IGHV mutation analysis, telomere length (TL) and CD49d expression. This observation was confirmed in an additional cohort of CLL patients treated with chemotherapy alone, derived from the CLL4 UK trial (Supplementary Table 3) [5]. Details of all the methods are given in the Supplementary materials.

**Fig. 1:**
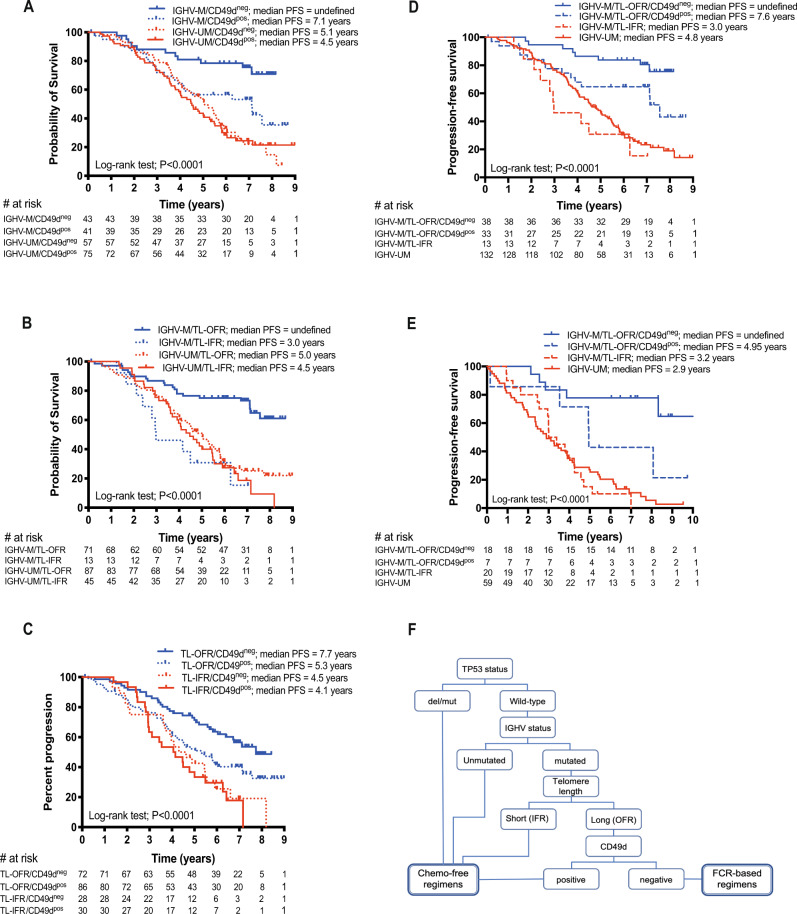
A risk-stratification algorithm for assigning frontline therapy in CLL using IGHV mutation status, telomere length and CD49d expression. Combined analyses of the pairs of biomarkers **A** IGHV mutation status and CD49d, **B** IGHV mutation status and telomere length and **C** telomere length and CD49d as predictors of progression-free survival (PFS) in CLL patients. **D** Shows the overlaid Kaplan–Meier curves for the ARCTIC/ADMIRE cohort, which demonstrate that patients with mutated IGHV genes and short telomeres have a similar, inferior PFS to the unmutated IGHV subset. Furthermore, patients with mutated IGHV genes, long telomeres and low CD49d expression have a significantly longer PFS than patients with mutated IGHV genes, long telomeres and high CD49d expression. **E** An additional cohort, derived from the FC-treated arm of the UK CLL4 trial, confirmed the findings from the ARCTIC/ADMIRE cohort. **F** Shows a schematic diagram of the propose a risk-adapted approach to treatment selection, which would contra-indicate frontline chemoimmunotherapy for ~83% of patients.

In the context of the ARCTIC/ADMIRE cohort, patients were first dichotomised according to TP53 disruption (Supplementary Fig. 2A) [2, 6], then according to IGHV gene status, TL and CD49d expression (Supplementary Fig. 3A–C). Samples were randomized for analysis of CD49d expression in our two centres; there was no difference in PFS between the two cohorts (Supplementary Fig. 4). Combining these three biomarkers in pairwise PFS analyses revealed differences in the prognostic impact of CD49d expression and TL in the context of mutated (M-IGHV) versus unmutated (UM-IGHV) cases suggesting the possibility of interactions between CD49d expression and/or TL with IGHV gene mutational status. In particular, according to bivariate Cox proportional hazard models, CD49d^pos^ cases in the M-IGHV subset associated with higher risk of disease progression compared to CD49d^neg^ cases (HR = 2.49 (95% CI, [1.19–5.18], *P* = 0.015), whilst no additional prognostic information was provided when a high CD49d expression was tested in the context of UM-IGHV CLL (HR = 1.07 [95% CI, [0.71–1.61], *P* = 0.76) (Fig. 1A).

Similarly, short telomere length (TL-IFR) associated with shorter PFS (HR = 4.29 [95% CI, 1.94–9.48], *P* = 0.0003) when compared with long telomere length (TL-OFR) in CLL patients with M-IGHV gene status, but not when tested in the context of UM-IGHV CLL (HR = 1.19 [95% CI, 0.78–1.80], *P* = 0.42) (Fig. 1B). Finally, despite the trend of the PFS curves indicating a detrimental effect of high CD49d expression in the TL-OFR group (HR = 1.73 [95% CI, 1.10–2.70], *P* = 0.017) but not in CLL patients with TL-IFR (HR = 1.09 [95% CI, 0.60–1.98], *P* = 0.78; Fig. 1C), no statistically significant interaction was documented between these variables (*P* = 0.20).

To take into account interactions between TL or CD49d with IGHV gene status, a multivariable model was generated to evaluate the independent prognostic impact of TL, CD49d expression and their combinations in the context of CLL with either UM-IGHV or M-IGHV gene status (Supplementary Table 4 and Fig. 1A–C). In detail, while neither TL nor CD49d expression had a prognostic impact in UM-IGHV CLL, the M-IGHV group could be significantly stratified by both TL (*P* = 0.002), and TL/CD49d combinations (*P* = 0.0014, Supplementary Table 4; *P* < 0.001, Fig. 1A–C). This data was used to develop the following hierarchical risk-stratification algorithm (Table 1 and Fig. 1D): (i) UM-IGHV CLL (132/216), single category no further dissected by TL and CD49d expression: 19.0% 8-year PFS (HR = 5.58 [3.70–8.42]); (ii) M-IGHV CLL with TL-IFR (13/84; 15.5%), no further dissected by CD49d expression (8-year PFS, 15.4%, HR = 6.45 [1.84–22.58]); this subset showed a median PFS similar to UM-IGHV CLL (3.0 versus 4.8 years, respectively, *P* = 0.29; Fig. 2A); (iii) M-IGHV CLL with TL-OFR (71/84); further stratified by CD49d expression (CD49dpos = 33/71; CD49dneg = 38/71) into categories with different PFS, i.e. 43.1% 8-year PFS (HR = 2.52 [1.08–5.89]) for CD49d^pos^ versus 75.5% 8-year PFS in the M-IGHV/TL-OFR/CD49d^neg^ reference group (Table 1 and Fig. 1D).

**Table 1:** PFS according to combination of IGHV gene mutational status, TL and CD49d expression.

IGHV	TL	CD49d	Patients	PFS^a^				HR (95% CI)^b^
				1 year	2 years	5 years	8 years	
ARCTIC-ADMIRE cohort								
UNMUT			132	96.2%	88.6%	47.0%	19.0%	5.58 (3.70–8.42)
MUT	TL-IFR		13	100%	84.6%	30.8%	15.4%	6.45 (1.84–22.58)
MUT	TL-OFR	Pos	33	93.8%	84.1%	64.7%	43.1%	2.52 (1.08–5.89)
MUT	TL-OFR	Neg	38	100%	94.6%	83.8%	75.5%	Reference
UK CLL4 cohort								
UNMUT			59	81.4%	66.1%	27.1%	5.4%	6.81 (4.04–11.46)
MUT	TL-IFR		20	90.0%	80.0%	15.0%	0.0%	6.27 (2.75–14.32)
MUT	TL-OFR	Pos	7	85.7%	85.7%	42.9%	21.4%	3.08 (0.73–13.02)
MUT	TL-OFR	Neg	18	100%	100%	77.8%	77.8%	Reference

*PFS* progression-free survival, *IGHV* immunoglobulin heavy chain variable, *TL* telomere length, *HR* hazard ratio, *CI* confidence interval, *UNMUT* UM-IGHV gene mutational status, *MUT* M-IGHV gene mutational status, *TL-ORF* telomere length outside fusogenic range, *TL-IFR* telomere length inside fusogenic range, *Neg* negative (i.e. CD49d < 30% of positive cells), *Pos* positive (i.e. CD49d ≥ 30% of positive cells).

^a^Estimated through the Kaplan–Meier method.

^b^Estimated from Cox proportional hazard model.

The proposed hierarchical model, by combining IGHV gene status, TL and CD49d expression, gave a Harrell’s C-index of 0.62 (0.56–0.67), which outperformed the predictive power of all the 2-predictor combinations, as well as that of any single predictor (*P* < 0.0001 in all comparisons; Supplementary Table 5). Similar results were obtained in an additional cohort of 104/119 TP53-wt CLL patients (Supplementary Fig. 2B), treated with FC in the UK CLL4 trial, where high CD49d expression, UM-IGHV genes and TL-IFR were, respectively, found in 52, 59 and 70 cases, all significantly associated with shorter PFS (see Supplementary Fig. 3D–F, Fig. 1E and Table 1).

Taken together, our analysis confirmed that TP53 disruption and IGHV mutation status are important determinants of response to C(I)T, but the addition of TL and CD49d expression into the prognostic stratification algorithm facilitated the identification of a group of patients who benefited most from FCR/FCR-based treatment regimens. These patients all had mutated immunoglobulin genes, long telomeres and low expression of CD49d (M-IGHV/TL-OFR/CD49d^neg^, Fig. 1F). Although this good prognosis group only constituted 17.5% of all cases in the combined cohorts (56/320 cases), they showed a long-term (8-year) PFS of more than 75% (Table 1). Furthermore, our data concur with the high 3-year PFS rate in M-IGHV patients treated with the FCR regimen in the E1912 study [7]. It is tempting to speculate that further stratification of the M-IGHV subset in the E1912 study would confirm that the M-IGHV/TL-OFR/CD49d^neg^ have a particularly good prognosis following FCR treatment [7].

In contrast, 82.5% of patients, i.e. 264/320 in the combined cohort, showed a sub-optimal response to C(I)T, indicating that this category of patients should be considered for alternative (chemo-free) regimens in the frontline setting (Fig. 1F). The importance of prospective identification of patients who would not benefit from CIT is highlighted by the fact that prior treatment history is one of the strongest adverse risk factors in predictive models of survival following ibrutinib treatment [8]. Conversely, given the recognized cardio-vascular toxicities associated with ibrutinib treatment [9], patients affected by hypertension, cardiac arrhythmias and/or high haemorrhagic risk could be redirected with more confidence toward CIT regimens if they were categorized as M-IGHV/TL-OFR/CD49d^neg^.

The prognostic model presented here more accurately defines which CLL with M-IGHV gene status will obtain the most benefit from CIT. In this regard, our analysis reinforces the potential utility of predictive biomarkers in treatment decision-making and extends and refines the results obtained from previous clinical trials [10], and a real-world retrospective multi-centre cohort [6], all with comparable or lower median follow-up than the cohorts investigated here. In Rossi et al. [6], the most benefit associated with FCR treatment was seen in CLL cases with M-IGHV concomitantly lacking TP53 disruption and 11q deletion. In the present study, we identified 77 cases bearing 11q deletion out of 320 cases (24%) from the composite ARCTIC/ADMIRE and CLL4 cohorts. Although 11q deletion is the sole prognosticator retaining significance along with our hierarchical model in multivariate analysis (Supplementary Table 6), the vast majority of 11q deleted cases had UM-IGHV gene status (61/77, 79%) or presented with the M-IGHV/TL-IFR phenotype (12/77, 16%). Consistently, of the CLL cases bearing an 11q deletion, only two were in the long-term PFS cases expressing the M-IGHV/TL-OFR/CD49d^neg^ phenotype (Supplementary Table 7).

Currently, the inclusion of TL evaluation in the proposed algorithm may be considered problematic as TL analysis has not yet been subjected to international standardisation. However, if a biomarker is deemed useful enough, then the requisite validation and standardisation will be performed as exemplified by the international effort to standardise IGHV mutation analysis, as well as TP53 mutations [11, 12]. As part of the effort to develop a robust, reliable and accurate TL assay, suitable for clinical applications, high-throughput STELA was developed; this assay circumvents the labour intensive and technical issues of the original single molecule STELA assay and it has been already successfully employed for the evaluation of large numbers of clinical samples [13]. As such, it offers significant advantages over all of the other currently available TL measuring methods.

Finally, from the biological point of view, there is a strong rationale for the combined power of the predictive tools proposed here. In particular, TP53 disruption is known to blunt the apoptotic response to drugs, UM-IGHV genes are associated with increased pro-survival/proliferative signals through B-cell receptors, short telomeres, by driving genomic complexity, have a role in cell survival and proliferation history [14], and, finally, CD49d plays a critical role in CLL cell adhesion, migration back to proliferation sites and cell survival [15]. The negative prognostic impact of high CD49d expression and short TL in the context of M-IGHV CLL rather than UM-IGHV cases, as revealed here by the statistically significant interactions of these prognostic markers, is consistent with their specific biological activities.

Given the favourable outcome of CLL patients with the M-IGHV/TL-OFR/CD49d^neg^ phenotype following treatment with CIT, assessment of whether novel agents provide additional benefit in this biological subgroup will require additional studies with long follow-up as well as an evaluation of quality of life and long-term toxicities. A further refinement of the proposed algorithm may be possible by incorporating information regarding the genetic abnormalities known to confer high/intermediate risk in CLL (e.g. BIRC3, NOTCH1 and SF3B1) [1]. These data are being generated in a parallel genetic analysis of the ARCTIC and ADMIRE cohorts.

## Supplementary information

Supplementary material

## References

[1] Hallek M, Cheson BD, Catovsky D, Caligaris-Cappio F, Dighiero G, Döhner H (2018). iwCLL guidelines for diagnosis, indications for treatment, response assessment, and supportive management of CLL. Blood.

[2] Fink AM, Böttcher S, Ritgen M, Fischer K, Pflug N, Eichhorst B (2013). Prediction of poor outcome in CLL patients following first-line treatment with fludarabine, cyclophosphamide and rituximab. Leukemia.

[3] Howard DR, Munir T, McParland L, Rawstron AC, Milligan D, Schuh A (2017). Results of the randomized phase IIB ARCTIC trial of low-dose rituximab in previously untreated CLL. Leukemia.

[4] Munir T, Howard DR, McParland L, Pocock C, Rawstron AC, Hockaday A (2017). Results of the randomized phase IIB ADMIRE trial of FCR with or without mitoxantrone in previously untreated CLL. Leukemia.

[5] Catovsky D, Richards S, Matutes E, Oscier D, Dyer M, Bezares RF (2007). Assessment of fludarabine plus cyclophosphamide for patients with chronic lymphocytic leukaemia (the LRF CLL4 Trial): a randomised controlled trial. Lancet.

[6] Rossi D, Terzi-di-Bergamo L, De Paoli L, Cerri M, Ghilardi G, Chiarenza A (2015). Molecular prediction of durable remission after first-line fludarabine-cyclophosphamide-rituximab in chronic lymphocytic leukemia. Blood.

[7] Shanafelt TD, Wang XV, Kay NE, Hanson CA, O’Brien S, Barrientos J (2019). Ibrutinib-rituximab or chemoimmunotherapy for chronic lymphocytic leukemia. N Engl J Med.

[8] Ahn IE, Tian X, Ipe D, Cheng M, Albitar M, Tsao LC, et al. Prediction of outcome in patients with chronic lymphocytic leukemia treated with ibrutinib: development and validation of a four-factor prognostic model. J Clin Oncol. 2021;39:576–85. 10.1200/JCO.20.00979 PMC8189626 33026937

[9] Lampson BL, Yu L, Glynn RJ, Barrientos JC, Jacobsen ED, Banerji V (2017). Ventricular arrhythmias and sudden death in patients taking ibrutinib. Blood.

[10] Thompson PA, Lévy V, Tam CS, Al Nawakil C, Goudot FX, Quinquenel A (2016). Atrial fibrillation in CLL patients treated with ibrutinib. An international retrospective study. Br J Haematol.

[11] Zalcberg I, D’Andrea MG, Monteiro L, Pimenta G, Xisto B. Multidisciplinary diagnostics of chronic lymphocytic leukemia: European Research Initiative on CLL-ERIC recommendations. Hematol Transfus Cell Ther. 2020;42:269–74. 10.1016/j.htct.2019.07.006 PMC7417454 31784406

[12] Malcikova J, Tausch E, Rossi D, Sutton LA, Soussi T, Zenz T (2018). ERIC recommendations for TP53 mutation analysis in chronic lymphocytic leukemia-update on methodological approaches and results interpretation. Leukemia.

[13] Norris K, Hillmen P, Rawstron A, Hills R, Baird DM, Fegan CD (2019). Telomere length predicts for outcome to FCR chemotherapy in CLL. Leukemia.

[14] Jebaraj BMC, Tausch E, Landau DA, Bahlo J, Robrecht S, Taylor-Weiner AN (2019). Short telomeres are associated with inferior outcome, genomic complexity, and clonal evolution in chronic lymphocytic leukemia. Leukemia.

[15] Zucchetto A, Benedetti D, Tripodo C, Bomben R, Dal Bo M, Marconi D (2009). CD38/CD31, the CCL3 and CCL4 chemokines, and CD49d/vascular cell adhesion molecule-1 are interchained by sequential events sustaining chronic lymphocytic leukemia cell survival. Cancer Res.

