# Supplementary material for: Combined analysis of IGHV mutations, telomere length and CD49d identifies long-term progression-free survivors in TP53 wild-type CLL treated with FCR-based therapies
Source: Leukemia. 2021 Jun 19;36(1):271–4. doi: 10.1038/s41375-021-01322-1 (PMC8727296; doi:10.1038/s41375-021-01322-1)
Supplement: Supplementary file 1 — Supplementary material [file 41375_2021_1322_MOESM1_ESM.docx]

**Combined analysis of IGHV mutations, Telomere length and CD49d identifies long-term progression-free survivors in TP53 wild-type CLL treated with FCR-based therapies**

Andrea G.S. Pepper^1*^, Antonella Zucchetto^2*^, Kevin Norris^3^, Erika Tissino^2^, Jerry Polesel^4^, Zarni Soe^5^, David Allsup^6^, Anna Hockaday^7^, Pei Loo Ow^7^, Peter Hillmen^8^, Andrew Rawstron^8^, Daniel Catovsky^9^, Pietro Bulian^2^, Riccardo Bomben^2^, Duncan M. Baird^3^, Christopher D. Fegan^3^, Valter Gattei^2**^, Chris Pepper^1,3**^

^1^University of Sussex, Brighton and Sussex Medical School, Brighton, United Kingdom. ^2^Clinical and Experimental Onco-Hematology Unit, Centro di Riferimento Oncologico di Aviano (CRO) IRCCS, Aviano, Italy. ^3^Division of Cancer & Genetics, Cardiff University, School of Medicine, Heath Park, Cardiff, United Kingdom. ^4^Unit of Cancer Epidemiology, Centro di Riferimento Oncologico di Aviano (CRO) IRCCS, Aviano, Italy. ^5^Zarni Soe, Leeds Teaching Hospital Trust, Leeds, United Kingdom. ^6^Hull York Medical School, University of Hull, Hull, United Kingdom. ^7^Clinical Trials Research Unit, Leeds Institute of Clinical Trials Research, University of Leeds, Leeds, United Kingdom. ^8^Section of Experimental Haematology, Leeds Institute of Medical Research at St James's, University of Leeds, Leeds, United Kingdom. ^9^Institute of Cancer Research, Sutton, United Kingdom.

**Methods**

**CLL patients**

The study included a cohort of 255 CLL samples from two UK trials, ARCTIC and ADMIRE (ARCTIC/ADMIRE cohort; demographics and characteristics of patients in Supplementary Table 2); the original studies comprised patients with known TP53 deletions but did not undertake TP53 mutation analysis prior to study entry although pre-treatment samples were stored for subsequent analysis^1,2^. Among these patients, 125 were randomized to receive standard dose FCR, 63 received fludarabine, cyclophosphamide, mitoxantrone, and low-dose rituximab and 67 received fludarabine, cyclophosphamide, mitoxantrone, and rituximab. There was no significant difference in progression-free survival (PFS) between the three treatment arms of the study (P=0.67; Supplementary Figure 1), so all subsequent analysis was performed on the combined cohort. The median follow-up was 77.5 months with 157 progressions and 76 deaths, all deaths were preceded by disease progression.

All 255 patients included in this study were evaluated for TP53 mutations, as detailed below. According to this analysis, 29 were found to be either TP53 deleted or TP53 mutated, and were therefore excluded, in line with the current guidelines^3^. As expected, these 29 CLL patients experienced a shorter PFS as compared to TP53 wild-type (wt) cases (Supplementary Figure 2A). An additional 10 cases, for whom TP53 mutation or IGHV mutation analysis was not available, were also excluded to give a final cohort of 216 TP53 wt CLL cases.

An additional cohort was composed of 119 CLL samples derived from patients randomised to receive FC from the UK CLL4 trial^4^ (CLL4 cohort; demographics and characteristics of patients in Supplementary Table 3); this cohort included TP53 deleted cases but was not originally tested for TP53 mutations. The median follow-up was 67·2 months with 99 progressions and 77 deaths. A subsequent analysis of TP53 mutations on archive materials, allowed to identify 15 patients as either TP53 mutated or deleted experiencing shorter PFS compared to TP53 wt cases (Supplementary Figure 2B). The exclusion of these cases allowed to reach a final cohort was of 104 TP53 wt cases.

All the clinical samples evaluated were taken at study entry and were obtained from the Blood Cancer UK funded UK CLL Trials Biobank, University of Liverpool (19/NW/0573, IRAS 269007) with informed consent in accordance with the declaration of Helsinki. The REMARK criteria for the evaluation of tumour prognostic markers were followed throughout this study (Supplementary Table 1).^5^

**CD49d expression**

CD49d was measured by flow cytometry. CLL samples were triple labelled with anti-CD19 PE-Cy7 and anti-CD5 APC or CD5 FITC mAbs and with anti-CD49d PE. In all cases, setup experiments with fluorescence minus one and appropriate isotype controls were performed to verify staining specificity, as reported previously.^6,7^ Samples were dichotomised as CD49d^pos^ and CD49d^neg^ based on the established 30% cut-off and/or the presence of CD49d bimodal expression. ^6-8^ The samples were randomly assigned for CD49d analysis in either Brighton (n=125) or Aviano (n=130); there was no significant difference in either the CD49d status or the median PFS in CD49d^pos^ and CD49d^neg^ subsets defined by the two centres, which suggests that the inter-laboratory implementation of the CD49d assay was robust (Supplementary Figure 4).

**Telomere length**

Telomere length (TL) was measured using the high-throughput STELA (Single Telomere Length Analysis) assay, as measured at chromosome 7q, and patients were bifurcated according to the previously established telomere dysfunction threshold.^9,10^ Patients were assigned as having short telomeres, i.e., inside the fusogenic range (TL-IFR), or long telomeres, i.e., outside the fusogenic range (TL-OFR).

**IGHV mutational status**

Amplifications of the IGHV-diversity(D)-joining(J) segment were performed from either reverse-transcribed total RNA or genomic DNA as previously reported.^11^ IGHV sequences were considered mutated (M-IGHV) or unmutated (UM-IGHV) using the conventional cutoff of 2% mismatch from germline IGHV sequences.^11,12^

**Mutational status of the *TP53* gene**

Analysis of *TP53* mutations was performed by NGS with an amplicon-based strategy, covering exons 2-11, in keeping with the ERIC recommendations.^13^ Specific primers were designed with the Primer3 program, and modified according to the Illumina (San Diego, CA) protocol by adding specific adapter sequences. Amplicon libraries were generated using a modified Illumina protocol starting from 40 ng of DNA (~6,000 diploid genomes). Multiplex PCR products were generated using Phusion High-Fidelity DNA Polymerase (Thermo Scientific, Milan, Italy) and subsequently tagged with specific index according to modified procedures for NexteraXT (DNALibrary Preparation kit, Illumina), as previously reported.^14,15^ Purified libraries were pooled and paired-end sequenced in a MiSeq instrument (Illumina). Results were analysed using the Miseq reporter software (Illumina). A specific CLL case was called as TP53 mutated if the variant allele frequency was equal or exceeded the 10% cutoff as per ERIC recommendations.^13^

**Statistical analysis**

Statistical analyses were carried out using SAS Software 9.4 and the open source R 3.6 (http://www.r-project.org/) statistical software. An a priori power analysis was conducted to estimate the minimum detectable effect size, given the available number of 216 patients and α=0.05. Assuming that approximately 60% of patients are in the group with the worse prognosis (progression rate during follow-up = 80%) and that 20% of patients are in the group with the best prognosis (progression rate = 25%), the study had a power of 85% to detect an HR of progression ≥2, using a Cox proportional hazards model. In univariate and multivariable analyses, all the individual prognostic markers were considered as categorical variables using previously established thresholds.^3,6,7,10^ PFS was chosen as the clinical readout of the present study in order to evaluate the long-term effect of CIT without considering other second line treatments, in keeping with previous studies^9,16-18^. For each patient, PFS was calculated from the date of treatment initiation to progression or last follow-up. Univariate comparisons for PFS were conducted using the log-rank test and displayed as Kaplan-Meier curves. The predictivity of each biomarker and their combination was evaluated through Harrell’s C-statistic.^19^ The statistical significance between Harrell’s C-indices was evaluated by applying the Student’s *t*-test and internally validated by applying a bootstrapping procedure.^20^ The hazard ratio (HR) for progression was estimated according to Cox proportional hazards models, conditioned on clinical trial. Interaction between prognostic markers was tested including the main effects and interaction terms in the Cox regression model. Association between variable was calculated using the chi-square test. In all cases P<0.05 (two-tailed) was considered significant.

**References**

1. Howard DR, Munir T, McParland L, et al. Results of the randomized phase IIB ARCTIC trial of low-dose rituximab in previously untreated CLL. *Leukemia*. 2017;31(11):2416-2425.

2. Munir T, Howard DR, McParland L, et al. Results of the randomized phase IIB ADMIRE trial of FCR with or without mitoxantrone in previously untreated CLL. *Leukemia*. 2017;31(10):2085-2093.

3. Zalcberg I, D'Andrea MG, Monteiro L, Pimenta G, Xisto B. Multidisciplinary diagnostics of chronic lymphocytic leukemia: European Research Initiative on CLL - ERIC recommendations. *Hematol Transfus Cell Ther*. 2019.

4. Catovsky D, Richards S, Matutes E, et al. Assessment of fludarabine plus cyclophosphamide for patients with chronic lymphocytic leukaemia (the LRF CLL4 Trial): a randomised controlled trial. *Lancet*. 2007;370(9583):230-239.

5. McShane LM, Altman DG, Sauerbrei W, Taube SE, Gion M, Clark GM: Reporting recommendations for tumor marker prognostic studies (REMARK). *J Natl Cancer Inst* 2005; 97: 1180-1184.

6. Gattei V, Bulian P, Del Principe MI, et al. Relevance of CD49d protein expression as overall survival and progressive disease prognosticator in chronic lymphocytic leukemia. *Blood*. 2008;111(2):865-873.

7. Majid A, Lin TT, Best G, et al. CD49d is an independent prognostic marker that is associated with CXCR4 expression in CLL. *Leuk Res*. 2011;35(6):750-756.

8. Tissino E, Pozzo F, Benedetti D, et al. CD49d promotes disease progression in chronic lymphocytic leukemia: new insights from CD49d bimodal expression. *Blood*. 2020;135(15):1244-1254.

9. Norris K, Hillmen P, Rawstron A, et al. Telomere length predicts for outcome to FCR chemotherapy in CLL. *Leukemia*. 2019;33(8):1953-1963.

10. Lin TT, Letsolo BT, Jones RE, et al. Telomere dysfunction and fusion during the progression of chronic lymphocytic leukemia: evidence for a telomere crisis. *Blood*. 2010;116(11):1899-1907.

11. Damle RN, Wasil T, Fais F, et al. Ig V gene mutation status and CD38 expression as novel prognostic indicators in chronic lymphocytic leukemia. *Blood*. 1999;94(6):1840-1847.

12. Hamblin TJ, Davis Z, Gardiner A, Oscier DG, Stevenson FK. Unmutated Ig V(H) genes are associated with a more aggressive form of chronic lymphocytic leukemia. *Blood*. 1999;94(6):1848-1854.

13. Malcikova J, Tausch E, Rossi D, et al. ERIC recommendations for TP53 mutation analysis in chronic lymphocytic leukemia-update on methodological approaches and results interpretation. *Leukemia*. 2018;32(5):1070-1080.

14. Pozzo F, Bittolo T, Vendramini E, et al. NOTCH1-mutated chronic lymphocytic leukemia cells are characterized by a MYC-related overexpression of nucleophosmin 1 and ribosome-associated components. *Leukemia*. 2017;31(11):2407-2415.

15. D'Agaro T, Bittolo T, Bravin V, et al. NOTCH1 mutational status in chronic lymphocytic leukaemia: clinical relevance of subclonal mutations and mutation types. *Br J Haematol*. 2018;182(4):597-602.

16. Rossi D, Terzi-di-Bergamo L, De Paoli L, et al. Molecular prediction of durable remission after first-line fludarabine-cyclophosphamide-rituximab in chronic lymphocytic leukemia. *Blood*. 2015;126(16):1921-1924.

17. Fischer K, Bahlo J, Fink AM, et al. Long-term remissions after FCR chemoimmunotherapy in previously untreated patients with CLL: updated results of the CLL8 trial. *Blood*. 2016;127(2):208-215.

18. Thompson PA,Tam CS, O'Brien SM, et al. Fludarabine, cyclophosphamide, and rituximab treatment achieves long-term disease-free survival in IGHV-mutated chronic lymphocytic leukemia. *Blood*. 2016;127(3):303-309.

19. International CLLIPIwg. An international prognostic index for patients with chronic lymphocytic leukaemia (CLL-IPI): a meta-analysis of individual patient data. *Lancet Oncol*. 2016;17(6):779-790.

20. Cohen JA, Rossi FM, Zucchetto A, et al. A laboratory-based scoring system predicts early treatment in Rai 0 chronic lymphocytic leukemia. *Haematologica*. 2019.

**Supplementary Table 1.** REMARK criteria

| **Item to be reported** | | **Page no.** |
| --- | --- | --- |
| **INTRODUCTION** | |  |
| 1 | State the marker examined, the study objectives, and any pre-specified hypotheses. | 3 |
| **MATERIALS AND METHODS** | |  |
| *Patients* | |  |
| 2 | Describe the characteristics (e.g., disease stage or co-morbidities) of the study patients, including their source and inclusion and exclusion criteria. | Tables S1 and S2 |
| 3 | Describe treatments received and how chosen (e.g., randomized or rule-based). | 3 |
| *Specimen characteristics* | |  |
| 4 | Describe type of biological material used (including control samples) and methods of preservation and storage. | 3-4 |
| *Assay methods* | |  |
| 5 | Specify the assay method used and provide (or reference) a detailed protocol, including specific reagents or kits used, quality control procedures, reproducibility assessments, quantitation methods, and scoring and reporting protocols. Specify whether and how assays were performed blinded to the study endpoint. | 4-5 |
| *Study design* | |  |
| 6 | State the method of case selection, including whether prospective or retrospective and whether stratification or matching (e.g., by stage of disease or age) was used. Specify the time period from which cases were taken, the end of the follow-up period, and the median follow-up time. | 3-5 |
| 7 | Precisely define all clinical endpoints examined. | 5 |
| 8 | List all candidate variables initially examined or considered for inclusion in models. | Tables S1 and S2 |
| 9 | Give rationale for sample size; if the study was designed to detect a specified effect size, give the target power and effect size. | Tables S1 and S2 |
| *Statistical analysis methods* | |  |
| 10 | Specify all statistical methods, including details of any variable selection procedures and other model-building issues, how model assumptions were verified, and how missing data were handled. | 5, Table S4 |
| 11 | Clarify how marker values were handled in the analyses; if relevant, describe methods used for cutpoint determination. | 5 |
| **RESULTS** | |  |
| *Data* | |  |
| 12 | Describe the flow of patients through the study, including the number of patients included in each stage of the analysis (a diagram may be helpful) and reasons for dropout. Specifically, both overall and for each subgroup extensively examined report the numbers of patients and the number of events. | 3-5 |
| 13 | Report distributions of basic demographic characteristics (at least age and sex), standard (disease-specific) prognostic variables, and tumor marker, including numbers of missing values. | Table S1 and S2 |
| *Analysis and presentation* | |  |
| 14 | Show the relation of the marker to standard prognostic variables. | 7-8 |
| 15 | Present univariable analyses showing the relation between the marker and outcome, with the estimated effect (e.g., hazard ratio and survival probability). Preferably provide similar analyses for all other variables being analyzed. For the effect of a tumor marker on a time-to-event outcome, a Kaplan-Meier plot is recommended. | 7-8 |
| 16 | For key multivariable analyses, report estimated effects (e.g., hazard ratio) with confidence intervals for the marker and, at least for the final model, all other variables in the model. | Tables 1 and 2 |
| 17 | Among reported results, provide estimated effects with confidence intervals from an analysis in which the marker and standard prognostic variables are included, regardless of their statistical significance. | 5-7 |
| 18 | If done, report results of further investigations, such as checking assumptions, sensitivity analyses, and internal validation. | 6-7, Table S4 |
| **DISCUSSION** | |  |
| 19 | Interpret the results in the context of the pre-specified hypotheses and other relevant studies; include a discussion of limitations of the study. | 7-9 |
| 20 | Discuss implications for future research and clinical value. | 8-9 |

Source: McShane LM, Altman DG, Sauerbrei W, Taube SE, Gion M, Clark GM: Reporting recommendations for tumor marker prognostic studies (REMARK). *J Natl Cancer Inst* 2005; 97: 1180-1184.

**Supplementary Table 2.** Characteristics of patients recruited into the UK trials ARCTIC and ADMIRE.

| **Parameter** | **Number** |
| --- | --- |
| Total number of CLL samples | 255 |
| Median age at study entry (range) | 63 (36-80) |
| Gender  Male  Female | 198  57 |
| Binet stage at study entry  A progressive  B  C | 38  126  91 |
| *IGHV*-mutated  *IGHV*-unmutated  ND | 99  153  3 |
| Telomere length  OFR  IFR | 175  80 |
| CD49^neg^ (<30%)  CD49^pos^ (≥30%)  ND | 115  139  1 |
| CD38^neg^ (<20%)  CD38^pos^ (≥20%)  ND | 151  102  2 |
| B2M (<3.5mg/L)  B2M (≥3.5mg/L)  ND | 60  177  18 |
| 11q-  ND | 49  6 |
| 17p-/TP53 mut  ND | 29  7 |

*IGHV*-mutated: >2% deviation from the germline immunoglobulin sequence

*IGHV*-unmutated: ≤2% deviation from the germline immunoglobulin sequence

Telomere length OFR: long telomeres outside the fusogenic range

Telomere length IFR: short telomeres inside the fusogenic range

B2M – beta 2 microglobulin

ND – not determined

**Supplementary Table 3.** Characteristics of patients recruited into the UK CLL4 trial and randomised to receive fludarabine plus cyclophosphamide.

| **Parameter** | **Number** |
| --- | --- |
| Total number of CLL samples | 119 |
| Median age at study entry (range) | 64  (43-83) |
| Gender  Male  Female | 84  35 |
| Binet stage at study entry  A progressive  B  C | 24  58  37 |
| *IGHV*-mutated  *IGHV*-unmutated | 46  73 |
| Telomere length  OFR  IFR | 37  82 |
| CD49^neg^ (<30%)  CD49^pos^ (≥30%) | 60  59 |
| CD38^neg^ (<20%)  CD38^pos^ (≥20%)  ND | 43  49  27 |
| B2M (<3.5mg/L)  B2M (≥3.5mg/L)  ND | 33  58  28 |
| 11q-  17p-/TP53 mut | 25  15 |

*IGHV*-mutated: >2% deviation from the germline immunoglobulin sequence

*IGHV*-unmutated: ≤2% deviation from the germline immunoglobulin sequence

Telomere length OFR: long telomeres outside the fusogenic range

Telomere length IFR: short telomeres inside the fusogenic range

B2M – beta 2 microglobulin

ND – not determined

**Supplementary Table 4.** HRs and corresponding CI for progression according to TL, CD49d expression, and IGHV gene mutation status. ADMIRE/ARCTIC cohort.

|  | **IGHV gene status** | | | | | |
| --- | --- | --- | --- | --- | --- | --- |
| **Variable** | **Unmutated** | | | **Mutated** | | |
|  | **Patients** | **Prog (%)** | **Multivariable**  **HR (95% CI)^a^** | **Patients** | **Prog (%)** | **Multivariable**  **HR (95% CI)^a^** |
|  |  |  |  |  |  |  |
| TL |  |  |  |  |  |  |
| TL-OFR | 87 | 69.8% | Ref. | 71 | 33.3% | Ref. |
| TL-IFR | 45 | 89.5% | 1.20 (0.79-1.82) | 13 | 87.9% | 3·60 (1.60-8.11) |
|  |  |  | P=0.40 |  |  | P=0.002 |
| CD49d |  |  |  |  |  |  |
| Neg | 57 | 78.2% | Ref | 43 | 37.8% | Ref |
| Pos | 75 | 80.5% | 1.09 (0.72-1.64) | 41 | 60.0% | 2.08 (0.98-4.42) |
|  |  |  | P=0.69 |  |  | P=0.058 |
| TL/CD49d | |  |  |  |  |  |
| TL-OFR /Neg | 34 | 69.1% | Ref | 38 | 23.2% | Ref. |
| TL-OFR /Pos | 53 | 70.4% | 1.15 (0.68-1.93) | 33 | 47.5% | 2.55 (1.07-6.09) |
| TL-IFR /Neg | 23 | 88.9% | 1.29 (0.70-2.39) | 5 | 83.3% | 6.46 (1.66-25.19) |
| TL-IFR /Pos | 22 | 89.8% | 1.29 (0.69-2.42) | 8 | 93.3% | 7.29 (2.53-21.00) |
|  |  |  | P=0.83 |  |  | P=0.0014 |
|  |  |  |  |  |  |  |

^a^Estimated from Cox proportional hazard model, conditioned on trial, and adjusted for IGHV, telomere length and CD49d.

HR, hazard ratio; CI, confidence interval; TL, telomere length; IGHV, Immunoglobulin heavy chain variable; Prog, progressed patients (%) at 8 years; TL-ORF, telomere length outside fusogenic range; TL-IFR, telomere length inside fusogenic range; Neg, negative (i.e., <30% of positive cells); Pos, positive (i.e., ≥30% of positive cells).

**Supplementary Table 5**. Harrell’s C-index and corresponding 95% confidence interval for different combinations of predictors of progression-free survival.

|  | **ARCTIC-ADMIRE cohort** | **CLL4 cohort** |
| --- | --- | --- |
|  |  |  |
| TL | 0.558 (0.519-0.597) | 0.621 (0.569-0.673) |
| CD49d | 0.554 (0.509-0.599) | 0.567 (0.514-0.620) |
| IGHV | 0.586 (0.539-0.632) | 0.602 (0.553-0.650) |
|  |  |  |
| TL, CD49d | 0.595 (0.549-0.641) | 0.634 (0.574-0.695) |
| TL, IGHV | 0.602 (0.552-0.653) | 0.644 (0.586-0.702) |
| CD49d, IGHV | 0.608 (0.555-0.660) | 0.615 (0.563-0.668) |
|  |  |  |
| TL, CD49d, IGHV***** | 0.616 (0.564-0.668) | 0.647 (0.587-0.708) |
|  |  |  |

*C-indices for the model containing three predictors were significantly higher (p<0.0001) than any other model including either two predictors or a single predictor.

TL, telomere length; IGHV, Immunoglobulin heavy chain variable.

**Supplementary Table 6.** Hazard ratio (HR) and corresponding confidence intervals (CI) for progression according to selected predictors in the combined ADMIRE/ARCTIC+CLL4 cohort.

| **Variable** | **Univariate** | | **Multivariable (n=291)** | |
| --- | --- | --- | --- | --- |
|  | **HR (95% CI)^a^** | **p-value** | **HR (95% CI)^b^** | **p-value** |
|  |  |  |  |  |
| Male gender (n=320) | 1.51 (1.07-2.15) | 0.0205 | 1.34 (0.91-1.97) | 0.1430 |
| Age (n=320) | 1.29 (0.98-1.69) | 0.0673 |  |  |
| Binet group (n=320) | 0.73 (0.52-1.03) | 0.0759 |  |  |
| B2M (n=281) | 1.37 (0.98-1.91) | 0.0625 |  |  |
| 11q23 deletion (n=309) | 2.40 (1.76-3.28) | <0.0001 | 1.66 (1.19-2.33) | 0.0029 |
| CD38 (n=293) | 1.39 (1.04-1.85) | 0.0258 | 0.97 (0.72-1.30) | 0.8191 |
| IGHV / TL / CD49d (n=320) |  |  |  |  |
| UNMUT | 6.78 (3.81-12.08) | <0.0001 | 6.94 (3.67-13.14) | <0.0001 |
| MUT / TL-IFR | 8.21 (4.17-16.15) | <0.0001 | 7.99 (3.83-16.69) | <0.0001 |
| MUT / TL-OFR / Pos | 2.97 (1.46-6.04) | 0.0038 | 3.95 (1.81-8.61) | 0.0006 |
| MUT / TL-OFR / Neg | Ref |  | Ref |  |
|  |  |  |  |  |

^a^Estimated from Cox proportional hazard model, conditioned on trial. ^b^Further adjusted for and adjusting variables significant in univariate analysis.

**Supplementary Table 7**. Characteristics of M-IGHV/TL-OFR/CD49d^neg^ CLL compared to the other categories.

|  | **M-IGHV/TL-OFR/CD49d^neg^** | **Other categories** | **P value*** |
| --- | --- | --- | --- |
| **Factor** | **n (%)** | **n (%)** |  |
| Age <65 yrs | 39 (69.6) | 147 (55.7) | 0.055 |
| ≥65 yrs | 17 (30.4) | 117 (44.3) |  |
| Sex male | 41 (73.2) | 208 (78.8) | 0.363 |
| female | 15 (26.8) | 56 (21.2) |  |
| Binet stage A | 6 (10.7) | 48 (18.2) | 0.191 |
| B | 26 (46.4) | 132 (50.0) |  |
| C | 24 (42.9) | 84 (31.8) |  |
| CD38 neg | 43 (76.8) | 127 (48.1) | 0.0004 |
| pos | 11 (19.6) | 112 (42.4) |  |
| ND | 2 (3.6) | 25 (9.5) |  |
| B2M <ULN | 18 (32.1) | 65 (24.6) | 0.375 |
| ≥ULN | 34 (60.7) | 164 (62.1) |  |
| ND | 4 (7.2) | 35 (13.3) |  |
| 11q del no | 53 (94.6) | 190 (72.0) | 0.0004 |
| yes | 2 (3.6) | 64 (24.2) |  |
| ND | 1 (1.8) | 10 (3.8) |  |

* according to Chi-square test

M-IGHV, mutated IGHV genes; TL-OFR, telomere length outside fusogenic range; CD49d^neg^, CD49d expression in <30% of CLL cells; yrs, years; CD38 neg, negative (expressed in <30% of CLL cells); CD38 pos, positive (expressed in ≥30% of CLL cells); ND, not determined; B2M, beta-2-microglobulin; ULN, upper limit of normal; 11q del, 11q deletion.

**Supplementary Figure 1.** Comparison of progression-free survival in patients randomized to receive fludarabine, cyclophosphamide, rituximab (FCR), fludarabine, cyclophosphamide, mitoxantrone, rituximab (FCMR), and fludarabine, cyclophosphamide, mitoxantrone, mini rituximab (FCM miniR) in the ARCTIC and ADMIRE clinical trials.

**Supplementary Figure 2.** **(A)** Comparison of progression-free survival (PFS) of TP53 wild-type and TP53 dysfunctional patients recruited into the ARCTIC and ADMIRE clinical trials. **(B)** Comparison of progression-free survival (PFS) of TP53 wild-type and TP53 dysfunctional patients who were randomized to receive FC in the UK CLL4 clinical trial. Consistent with previous studies, TP53 dysfunction was associated with significantly shorter PFS.

**Supplementary Figure 3.** The ability of CD49d, IGHV mutation status and telomere length to predict progression-free survival (PFS) in CLL patients. **(A)** CD49d **(B)** IGHV mutation status and **(C)** telomere length, were all able to identify subsets of patients with different PFS following treatment with FCR-based regimens (ARCTIC/ADMIRE cohort). An additional cohort, derived from the FC-treated arm of the UK CLL4 trial, gave similar results with **(D)** CD49d **(E)** IGHV mutation status and **(F)** telomere length all showing predictive power.

**Supplementary Figure 4.** Comparison of progression-free survival (PFS) in patients analyzed for CD49d in two centres, Brighton and CRO, Aviano. There was no significant difference in either the frequency of samples assigned as CD49d^pos^ and CD49d^neg^ between the two centres or in terms of PFS.
